# Comparative overview of innovation and patent filings in Radiopharmacy

**DOI:** 10.31744/einstein_journal/2020GS4816

**Published:** 2019-11-08

**Authors:** Ana Cláudia Camargo Miranda, Denise Rahal Lobato, Elaine Bortoleti de Araújo

**Affiliations:** 1 Hospital Israelita Albert Einstein, São Paulo, SP, Brazil.; 2 Instituto de Pesquisas Energéticas e Nucleares, São Paulo, SP, Brazil.

**Keywords:** Intellectual Property, Patent, Innovation, Radiopharmaceuticals, Nuclear medicine

## Abstract

**Objective:**

To expose the current situation of the Brazilian Nuclear Medicine in relation to innovation, taking into account the Intellectual Property protection and the particularities of this field.

**Methods:**

The number and the origin of patents filings from Brazil, United States and European Patent Convention countries were retrospectively compared in a 20-year period.

**Results:**

The number of accumulated patents filings of conventional pharmaceuticals was ten times higher compared to the radiopharmaceuticals in the three regions studied.

**Conclusion:**

The largest number of Brazilian patents filings corresponded to the international patent applications, which is related to the country development conditions, as well as to the difficulties in the process of patent filing.

## INTRODUCTION

The world experiences a moment of economic development based on the capacity of countries to generate and appropriate the technical-scientific knowledge to produce resources. This context provides a situation of competitiveness among the companies and promotes a constant search for new inventions and innovations, demonstrating the dislocation from the act of making to the act of knowing. Within this scenario, the issue of Intellectual Property protection becomes important as a mechanism to guarantee the rights of the inventor and promote investments in research.^1 - 3^

According to the World Intellectual Property Organization (WIPO), the Intellectual Property refers to the creation of the mind, defined as the result of human intellect that can be expressed in two categories: industrial property, which includes, among others, the patents (inventions); and copyrights, which include representations, that is, the materialization of ideas.^4^

**Figure 1:**
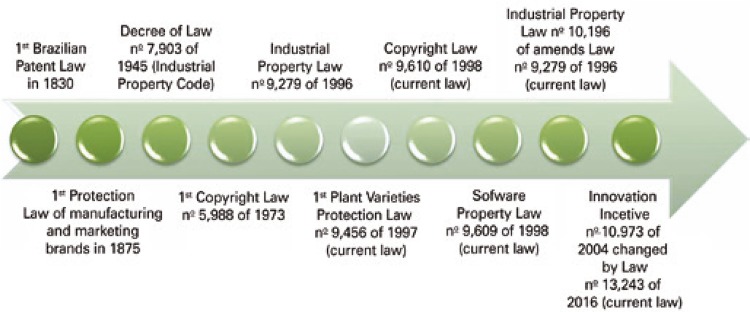
Timeline according to the creation of Brazilian laws pertaining to Intellectual Property

Protection of industrial property in Brazil initiated with issuance of the license on April 28, 1809. Brazil is classified as the fifth country of the world to establish the protection of the rights of the inventor. This mark fostered the industrial development of the time, establishing the era of Capitalism. To demonstrate the concern of the country in guaranteeing the respect of industrial property and the copyright, successive laws were created as of 1830 ( Figure 1 ).^5^

The intention of Intellectual Property protection is to assure the invention to the inventor, encouraging the development of new creations, in addition to fostering innovation and economic growth of a country, attracting industries and giving opportunity of new jobs for the population.^4^

One of the primary forms of Intellectual Property protection occurs by means of patent filing. The patent is an official temporary property title regarding an invention or model of use, granted by the State, by force of law, that confers to its titleholders or their successors the right to impede third parties from reproducing the object of the patent without their consent.^6 - 8^

In this respect, the pharmaceutical industry is one of the fields that have a great interest in the issue of Intellectual Property protection. This form of protection is, for the most part, directed towards the filing of patents, with the purpose of guaranteeing financial return on investments made relative to research and development of the registration of the drug and of its introduction in the market.^9^

Among the pharmaceutical sciences, Radiopharmacy has the particularity of developing, producing, and distributing or marketing drugs that contain a radioactive element (radiopharmaceuticals), utilized in diagnostic and therapeutic procedures in Nuclear Medicine. In Brazil, this area is governed by rules of radioprotection elected by the *Comissão Nacional de Energia Nuclear* (CNEN) [National Commission of Nuclear Energy] and by health regulations determined by the *Agência Nacional de Vigilância Sanitária* (ANVISA) [National Health Surveillance Agency].^10^ Issues of Intellectual Property protection also affect the radiopharmaceutical industry, although the development of new radiopharmaceuticals is less extensive, when compared to the conventional pharmaceutical industry. A new radiopharmaceutical, as is true with a new drug, should be registered in the country seeking to guarantee its quality, safety, and efficacy, as per the *Resolução da Diretoria Colegiada* (RDC) [Collegiate Resolution] number 64, of 2009, in public consultation at the moment.^11^

When the objective is to patent an invention or model of use in radiopharmaceuticals, one should take into consideration Law 9,279/96, which presents as non-patentable, among others, the substances, materials, mixtures, elements, or products of any type, as well as the modification of the physical-chemical properties and the respective process of acquisition or modification, when resulting from transformation of the atomic nucleus. This includes as non-patentable the radioactive elements, also called radionuclides or radioisotopes, produced from the nuclear transformation of stable nuclei. Radioisotopes represent the primary raw material used in the production of radiopharmaceuticals.^8^

In Brazil, the CNEN authorities, including the *Instituto de Pesquisas Energéticas e Nucleares* (IPEN) [Institute for Energy and Nuclear Research], *Instituto de Engenharia Nuclear* (IEN) [Nuclear Engineering Institute], *Centro de Desenvolvimento da Tecnologia Nuclear* (CDTN) [Nuclear Technology Development Center], and the *Centro Regional de Ciências Nucleares do Nordeste* (CRCN) [Regional Center of Nuclear Sciences of the Northeast Region] are references in radiopharmacy, since the production of radioisotopes is a monopoly of the Federal Government. With exception of production, marketing, and use of short half-life radioisotopes, for medical, agricultural, and industrial uses, the monopoly was partially broken up in 2006, with Constitutional Amendment 49.^10 , 12^

On the other hand, according to Law 10,196/01, the concession of a pharmaceutical patent in reference to products and processes, should, primarily, have the agreement of ANVISA, to then be directed to the *Instituto Nacional da Propriedade Industrial* (INPI) National Institute of Industrial Property.^13^ Besides observing the requirements for patentability, with this law the Health Surveillance Agency (ANVISA) aims to verify if the granting of a certain patent can imply limitation of access of the patients to the medicine under consideration.^9^

## OBJECTIVE

To show the current situation of the Brazilian Nuclear Medicine as to innovation, specifically comparing the number and origin of patent filings in Brazil, in the United States, and in countries participating in the European Patent Convention related to Radiopharmacy.

## METHODS

A literature review was done covering the theme of Intellectual Property and its forms of protection, based on the Brazilian laws, books, manuals, scientific articles, and public and private search engines, such as INPI, Espacenet Patent Search, Google Patents, Thomson Innovation, European Patent Office (EPO), and Questel Orbit^©^. From here the data survey was directed towards the processes that involve the two families of patents, A61K51/00 (preparations containing radioactive substances for use in therapy or testing *in vivo* ) and A61K49/00 (preparations for testing *in vivo* ), added to the key words “radiopharma” OR “radioisotope” OR “radioimmun” OR “radioactive” OR “radiotherap” OR “radiolabel” OR “radionuclide”, as well as from the family A61K31/00 (medicinal preparations containing organic active ingredients), with and without the addition of the keywords “radiographic contrast agent” OR “radiocontrast agents” OR “contrast imaging” OR “diagnostic imaging”. The purpose was to comparatively assess the number of patents filings in Brazil, in the United States, and in participating countries of the European Patent Convention (EPC), during the period of 20 retroactive years (1997 to 2017), and to identify the origin of the ten largest patent applicants.

## RESULTS

**Figure 2:**
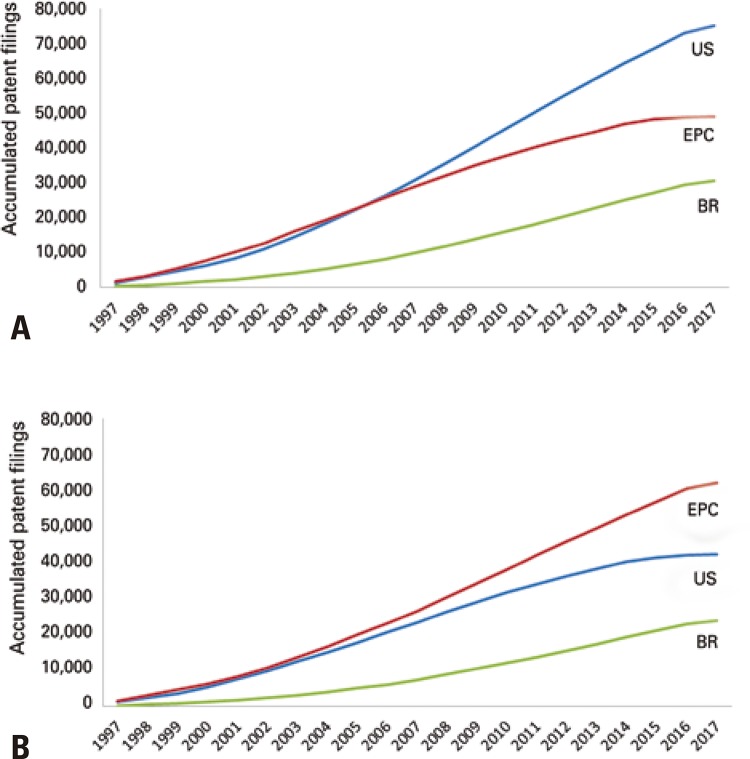
Comparison between the accumulated patent filings in Brazil (BR), in the United States (US), and in participating countries of the European Patent Convention (EPC), from 1997 to 2017. The search was performed by adding the keywords “radiopharma” OR “radioisotop” OR “radioimmun” OR“ radioactive” OR“ radiotherap” OR “radiolabel” OR “radionuclide”, by the Questel Orbit© software, in which (A) refers to family A61K51/00, and (B) to family A61K49/00

The specific search utilizing the Questel Orbit^©^ software enabled obtaining the results shown in figure 2 , in reference to families A61K51/00 (2A) and A61K49/00 (2B), added to the keywords “radiopharma” OR “radioisotope” OR “radioimmun” OR “radioactive” OR “radiotherap” OR “radiolabel” OR “radionuclide”.

**Figure 3:**
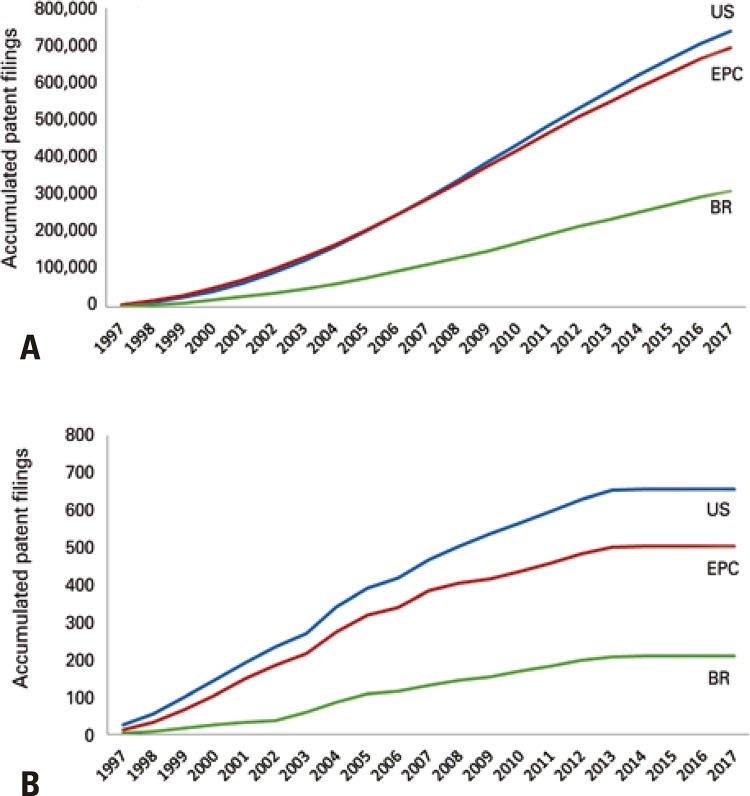
Comparison between the accumulated deposits of patents in Brazil (BR), United States (US) and in participating countries of the European Patent Convention (EPC), from 1997 to 2017, by the Questel Orbit© software, in reference to family A61K31/00. (A) Search not adding keywords. (B) Search adding the keywords “radiographic contrast agent” OR “radiocontrast agents” OR “contrast imaging” OR “diagnostic imaging”

To evaluate the tendency of patents filings in the area of conventional pharmaceuticals, a search was made for the A61K31/00 family (medicinal preparations containing organic active ingredients), presented in figure 3 , in which (3A) represents the results of the family without the addition of keywords, and (3B) of the search plus the keywords “radiographic contrast agent” OR “radiocontrast agents” OR “contrast imaging” OR “diagnostic imaging”.

**Figure 4:**
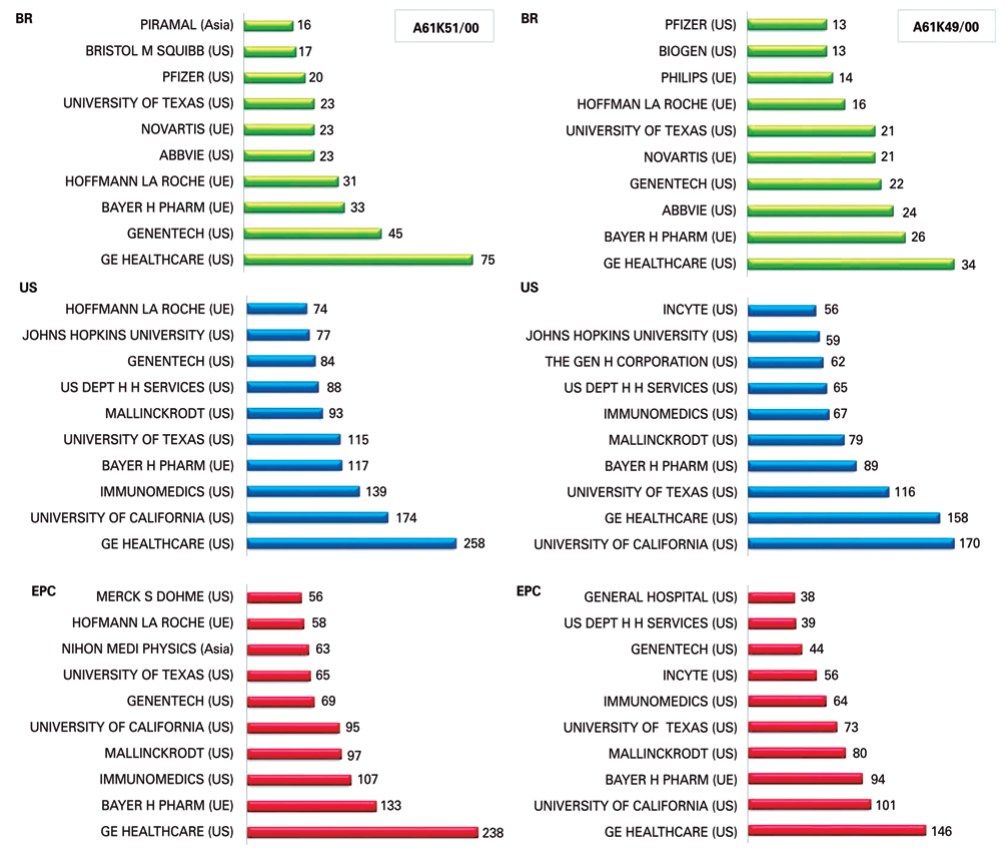
Comparison among the ten companies that filed more patents in Brazil (BR), in the United States (US), and in participating countries of the European Patent Convention (EPC), from 1997 to 2017, in reference to the families, A61K51/00 and A61K49/00 added to the keywords “radiopharma” OR “radioisotop” OR “radioimmun” OR “radioactive” OR “radiotherap” OR “radiolabel” OR “radionuclide” *.* This was a search conducted by the Questel Orbit© software

Figure 4 displays the main companies and organizations that filed patents in the families related to radiopharmaceutical preparations.

## DISCUSSION

In family A61K51/00 *,* the predominance of the United States in filing patents was noted, with marked growth over the last 10 years. Just as in United States, the number of patents filed in the EPC countries is higher than the patents filed in Brazil, but EPC presented with a drop in the pace of growth over the last 5 years, characterized by the tendency to plateau. The total number of patents accumulated by Brazil in this category during the studied period was 30,693, whereas in EPC countries and the United States it was 48,933 and 74,946, respectively. The number of accumulated patents registered in the United States in this category is approximately twice as high than that registered in Brazil.

As to the accumulated filings of patents in reference to the A61K49/00 family, there are more accumulated patents filed by EPC countries, followed by the United States and by Brazil. While the A61K51 family seems to better represent the patents related to radiopharmaceutical products, including the products that obtained registry with the regulatory health agencies, the A61K49 family is apparently associated, in general, to the experimental compounds, with a potential for application *in vivo* . In this family, similarly to what was observed for the category represented in figure 2A, the decreased pace of growth over the last few years draws attention, with a tendency to plateau.

In the A61K31/00 family, which compiles the data on conventional (non-radioactive) pharmaceuticals, the number of accumulated patent filings was ten times greater than that of families related to the processes involving radiopharmaceuticals; this is also the case in Brazil, with approximately 310,095 filed patents. The number of patent filings accumulated by the United States (738,891) is close to that of EPC countries deposits at (693,594), in the observation period. Additionally, in this family investigated, the pace was increasing, without the characterization of a plateau.

On the other hand, results of the A61K31/00 classification, which is restricted to pharmaceuticals like radiological contrast agents, used in imaging diagnoses, tend towards saturation in the United States, in Brazil, and in countries of the EPC.

When comparing accumulated patent filings in Brazil and in other regions included in this study, the number of filings was lower in Brazil, in all the families.^14^ Such a reality is likely a reflection, in large part, of the general level of development of the country, when compared to the United States and to the countries of the EPC, since Brazil relies on limited investments on the part of government and of the private sector for the development of new drug, including radiopharmaceuticals. Also, there are factors directly related to the Brazilian patent process: lack of support for researchers in the preparation process of patents, and cultural issues related to the incentive to the protection of the Intellectual Property generated. These issues can contribute to discourage Brazilian researchers to file patents. Another factor to be considered refers to the difficulties found in the patent process and the appreciation of a patent by the INPI. This aspect was considered in Resolution/INPI/PR 217, of May 3^rd^, 2018, which altered Resolution 80/2013 with the objective of controlling the priorization of the examination of patent applications and patents of products and pharmaceutical processes, as well as of equipment and materials related to public health.^15 , 16^

The small number of patent filings related to the radiopharmaceutical area in Brazil could be justified by the fact of the field being small in the country, when compared to that of pharmaceuticals in general; by the restricted powers of CNEN for the production and marketing of long half-life radiopharmaceuticals; and by the relatively recent opening for private enterprise, after the partial break up of the monopoly for production and marketing of short half-life radiopharmaceuticals (less than 2 hours).^12^ Additionally, the number of researchers dedicated to the radiopharmaceutical area in Brazil is considerably reduced. Despite such constrained characteristics, curiously, it is possible to confirm that the number of filings accumulated of Brazilian patents related to Radiopharmacy is ten times smaller than in the area of conventional pharmaceuticals – and this proportionality is similar to that noted in more developed countries.

The investigation of new drugs requires high investments to finance all the phases of development, from the design of the molecule or active ingredient, passing through the pharmacotechnical process and the methodologies used in quality control of the finished product, to Phases I to IV of non-clinical and clinical studies.^17^ The development cycle of new radiopharmaceuticals is identical to that of pharmaceuticals in general. Despite the fact that the market for new radiopharmaceuticals is small when compared to that of conventional pharmaceuticals, costs are comparable. This fact explains the lower number of patent filings in the radiopharmaceutical area when compared to that of medications in general, both in Brazil and in other countries.^18^

The health regulation for the production and registry of radiopharmaceuticals is relativly recent in Brazil, and was implemented in 2009, as of the publication of RDC 63, currently RDC 301/2019, and RDC 64/2009, in public consultation, for Good Manufacturing Practices and Registry of Radiopharmaceutical Products, respectively.^11 , 19^ Such regulations have become necessary, especially when one considers the break up of the production monopoly of radiopharmaceuticals in 2006, and the entrance of private enterprise in the market of radiopharmaceuticals.^12^

Nevertheless, RDC 64, which describes the need for conducting non-clinical and clinical studies for the registry of an innovative radiopharmaceutical, imposes rules similar to those adopted in the registry of conventional pharmaceuticals to this category of medicines, in order to guarantee the efficacy and safety of the product. Regulation requirements of this level are also observed in different countries and certainly collaborate to restrain the development and launching of new radiopharmaceuticals in the world stage, as the cost of this process increases a lot.^20 - 22^ The public consultation will enable to change the requirements for non-clinical and clinical studies.

If, on the one hand, the development of new drugs, for the most part, counts on the investment of large pharmaceutical companies that use patents to protect the capital invested, in the case of radiopharmaceuticals, in most countries, development is usually carried out in university research centers or research institutes for local production and use, on a small scale and with difficulty in guaranteeing the protection of the patent of the new radiopharmaceutical. On the other hand, the transfer of technology to constituted production companies seems to be an ever increasing reality in the area of Radiopharmacy.^23 , 24^

Finally, the ten foremost companies and organizations that deposited patents in the families investigated relative to the preparations of the radiopharmaceuticals had results that placed them as the ones responsible for the largest number of patent filings, despite the contribution of some universities - probably as an effect of the high costs involved in the investigation of new drugs.^25^ Patent filings in Brazil do not reflect internal development, but the tendency of the market, that is, the commercial interest of the international company, especially of American origin, taking into consideration the cost-benefit for each given innovation.

## CONCLUSION

The growing tendency of the countries to generate technical and scientific knowledge, each of them at their own pace of development, leads to the need to guarantee their rights and foster investments in a certain area. Thus, it is possible to visualize the importance of an investigator being assertive in the choice and conduction of experimental studies, aiming at innovative processes.

The greatest number of patent filings in Brazil related to the radiopharmaceutical area corresponds to the applications of international patents via the Patent Cooperation Treaty, in the same way as patent filings of conventional pharmaceuticals and/or contrast media for radiodiagnosis. This scenario is a reflection of the economic and scientific development conditions of the country, as well as how the difficulties presented in the patent filing process, showing that the investment in programs that facilitate the process and bring closer the research institutes and private universities, encouraging patent filing, in general, and innovation is indispensable.
