# Supplementary material for: Comparative overview of innovation and patent filings in Radiopharmacy
Source: Einstein (Sao Paulo). 2019 Nov 8;18:eGS4816. doi: 10.31744/einstein_journal/2020GS4816 (PMC6896597; doi:10.31744/einstein_journal/2020GS4816)
Supplement: Translation

# Translation: Panorama comparativo da inovação e do depósito de patentes em Radiofarmácia

**Authors:** Ana Cláudia Camargo Miranda, Denise Rahal Lobato, Elaine Bortoleti de Araújo

**Affiliations:** 1 Hospital Israelita Albert Einstein, São Paulo, SP, Brasil.; 2 Instituto de Pesquisas Energéticas e Nucleares, São Paulo, SP, Brasil.

**Keywords:** Propriedade Intelectual, Patente, Inovação, Compostos radiofarmacêuticos, Medicina Nuclear

## Abstract

**Objetivo:**

Evidenciar a situação atual da Medicina Nuclear brasileira em relação à inovação, considerando a proteção à Propriedade Intelectual e suas particularidades na área.

**Métodos:**

Foi realizado um levantamento comparativo do número e da origem de depósitos de patentes relacionadas a essa área no Brasil, nos Estados Unidos e em países participantes da *European Patent Convention* , em um período de 20 anos retroativos.

**Resultados:**

O número de depósitos acumulados de patentes de medicamentos convencionais foi dez vezes maior quando comparado às famílias relacionadas aos processos que envolvem radiofármacos, nas três regiões estudadas.

**Conclusão:**

O maior número de depósitos de patentes brasileiras correspondeu aos pedidos de patentes internacionais, refletindo as condições de desenvolvimento do país, bem como as dificuldades encontradas no processo de depósito de uma patente.

## INTRODUÇÃO

O mundo experimenta um momento de desenvolvimento econômico baseado na capacidade dos países gerarem e apropriarem-se do conhecimento técnico-científico para produzir recursos. Esse contexto proporciona um cenário de competitividade entre as empresas e promove a constante busca por novas invenções e inovações, evidenciando o deslocamento do ato de fazer para o ato de saber. Neste cenário, a questão da proteção à Propriedade Intelectual torna-se importante como mecanismo para garantir os direitos do inventor e promover os investimentos em pesquisa.^1 - 3^

Segundo a *World Intellectual Property Organization* (WIPO), a Propriedade Intelectual refere-se à criação da mente, definindo-se como o resultado do intelecto humano, que pode ser expresso em duas categorias: propriedade industrial, que inclui, entre outros, as patentes (invenções); e os direitos autorais, que abrangem as representações, ou seja, a materialização de ideias.^4^

A proteção à propriedade industrial no Brasil iniciou-se com a emissão do alvará de 28 de abril de 1809, sendo classificado como o quinto país do mundo a estabelecer a proteção aos direitos do inventor. Esse marco estimulou o desenvolvimento industrial da época, instituindo a era do capitalismo. Para evidenciar a preocupação do país em garantir o respeito à propriedade industrial e ao direito autoral, uma sucessão de leis foram criadas a partir de 1830 ( Figura 1 ).^5^

**Figura 1:** Linha do tempo conforme criação das leis brasileiras relacionadas à Propriedade Intelectual

O intuito da proteção à Propriedade Intelectual é o de assegurar a invenção ao inventor, incentivando o desenvolvimento de novas criações, além de promover a inovação e o crescimento econômico de um país, atraindo indústrias e promovendo a oportunidade de novos empregos à população.^4^

Uma das principais formas de proteção à Propriedade Intelectual ocorre por meio do depósito de patente. A patente é um título de propriedade temporário oficial sobre uma invenção ou modelo de utilidade, concedido pelo Estado, por força de lei, que confere ao seu titular ou seus sucessores o direito de impedir que terceiros reproduzam o objeto da patente sem seu consentimento.^6 - 8^

Nesse âmbito, a indústria farmacêutica é um dos ramos que possui grande interesse nas questões de proteção à Propriedade Intelectual. Essa forma de proteção, em sua maioria, é direcionada ao depósito de patentes, com vistas a garantir o retorno financeiro dos investimentos relacionados à pesquisa e ao desenvolvimento do registro do medicamento e de sua introdução no mercado.^9^

Dentre as vertentes da farmácia, encontra-se a Radiofarmácia, que é uma área das ciências farmacêuticas que apresenta a particularidade de desenvolver, produzir e distribuir ou comercializar fármacos que possuem em sua constituição um elemento radioativo (radiofármacos), utilizados em procedimentos diagnósticos e terapêuticos em medicina nuclear. No Brasil, essa área é regida por normas de radioproteção eleitas pela Comissão Nacional de Energia Nuclear (CNEN) e por normas sanitárias determinadas pela Agência Nacional de Vigilância Sanitária (ANVISA).^10^ As questões de proteção à Propriedade Intelectual também permeiam a indústria radiofarmacêutica, apesar do desenvolvimento de novos radiofármacos ocorrer em menor escala quando comparado à indústria farmacêutica convencional. Um novo radiofármaco, assim como um fármaco, deve ser registrado no país visando garantir sua qualidade, segurança e eficácia, conforme a Resolução da Diretoria Colegiada, a qual, no momento, encontra-se aberta à consulta pública.^11^

Quando se objetiva patentear uma invenção ou modelo de utilidade na área radiofarmacêutica, deve-se levar em consideração a lei 9.279/96, que apresenta como não patenteáveis, entre outros, as substâncias, matérias, misturas, elementos ou produtos de qualquer espécie, bem como a modificação de suas propriedades físico-químicas e os respectivos processos de obtenção ou modificação, quando resultantes de transformação do núcleo atômico. Isto inclui como não patenteáveis os elementos radioativos também denominados radionuclídeos ou radioisótopos, produzidos a partir da transformação nuclear de núcleos estáveis. Os radioisótopos representam a principal matéria-prima empregada na produção de radiofármacos.^8^

No Brasil, as autarquias da CNEN, incluindo o Instituto de Pesquisas Energéticas e Nucleares (IPEN), o Instituto de Engenharia Nuclear (IEN), o Centro de Desenvolvimento da Tecnologia Nuclear (CDTN) e o Centro Regional de Ciências Nucleares do Nordeste (CRCN), são as referências em Radiofarmácia, uma vez que a produção de radioisótopos é monopólio da União. Com exceção da produção, da comercialização e da utilização de radioisótopos de meia-vida curta, para usos médicos, agrícolas e industriais, o monopólio que foi parcialmente quebrado, em 2006, com a Emenda Constitucional 49.^10 , 12^

Por outro lado, segundo a lei 10.196/01, a concessão de uma patente farmacêutica referente a produtos e processos, deve, primeiramente, ter a anuência da ANVISA para, depois, ser direcionada ao Instituto Nacional da Propriedade Industrial (INPI).^13^ Além de observar os requisitos de patenteabilidade, o órgão da ANVISA visa, com essa lei, verificar se a concessão de determinada patente pode implicar na limitação do acesso dos pacientes ao medicamento em questão.^9^

## OBJETIVO

Evidenciar a situação atual da área Medicina Nuclear brasileira em relação à inovação, comparando especificamente o número e a origem de depósitos de patentes nacionais e internacionais.

## MÉTODOS

Foi realizada uma revisão bibliográfica abordando o tema da Propriedade Intelectual e suas formas de proteção, tendo como base a legislação brasileira, livros, manuais, artigos científicos e ferramentas de busca públicas e privadas, como INPI, *Espacenet Patent Search* , *Google Patents* , *Thomson Innovation, European Patent Office* (EPO) e *Questel Orbit* ^©^. A partir disso, o levantamento de dados foi direcionado aos processos que envolvem as famílias de patentes A61K51/00 ( *preparations containing radioactive substances for use in therapy or testing in vivo* ) e A61K49/00 ( *preparations for testing in vivo* ), acrescidas das palavras-chaves “ *radiopharma* ” *OR* “ *radioisotope* ” *OR* “ *radioimmun* ” *OR* “ *radioactive* ” *OR* “ *radiotherap* ” *OR* “ *radiolabel* ” *OR* “ *radionuclide* ”, e da família A61K31/00 ( *medicinal preparations containing organic active ingredients* ), com e sem o acréscimo das palavras-chaves “ *radiographic contrast agent* ” *OR* “ *radiocontrast agents* ” *OR* “ *contrast imaging* ” *OR* “ *diagnostic imaging* ”. O intuito foi avaliar comparativamente o número de patentes depositadas no Brasil, nos Estados Unidos e países participantes da Convenção Europeia de Patentes (CEP), no período de 20 anos retroativos (1997 a 2017), bem como identificar a origem dos dez maiores depositantes.

## RESULTADOS

A busca específica utilizando o programa Questel Orbit^©^proporcionou a obtenção dos resultados apresentados na figura 2 , referentes às famílias A61K51/00 (2A) e A61K49/00 (2B), acrescidas das palavras-chaves: “ *radiopharma* ” *OR* “ *radioisotope* ” *OR* “ *radioimmun* ” *OR* “ *radioactive* ” *OR* “ *radiotherap* ” *OR* “ *radiolabel* ” *OR* “ *radionuclide* ” *.*

**Figura 2:** Comparação entre os depósitos acumulados de patentes no Brasil (BR), nos Estados Unidos (EU) e em países participantes da Convenção Europeia de Patentes (CEP), no período de 1997 a 2017. Busca acrescida das palavras-chaves: “ *radiopharma* ” *OR* “ *radioisotop* ” *OR* “ *radioimmun* ” *OR* “ *radioactive* ” *OR* “ *radiotherap* ” *OR* “ *radiolabel* ” *OR* “ *radionuclide* ”, pelo programa Questel Orbit©, sendo (A) referente à família A61K51/00 e (B) à família A61K49/00

Para avaliar a tendência de depósito de patentes na área de medicamentos em geral, realizou-se a busca para a família A61K31/00 ( *medicinal preparations containing organic active ingredients* ), apresentada na figura 3 , sendo (3A) representativa dos resultados da família sem o acréscimo de palavras-chaves e (3B) da busca acrescida das palavras-chaves “ *radiographic contrast agent* ” *OR* “ *radiocontrast agents* ” *OR* “ *contrast imaging* ” *OR* “ *diagnostic imaging* ” *.*

**Figura 3:** Comparação entre os depósitos acumulados de patentes no Brasil (BR), nos Estados Unidos (EU) e em países participantes da Convenção Europeia de Patentes (CEP), no período de 1997 a 2017, pelo programa Questel Orbit©, referentes à família A61K31/00. (A) Busca sem o acréscimo de palavras-chaves. (B) Busca acrescida das palavras “ *radiographic contrast agent* ” *OR* “ *radiocontrast agents* ” *OR* “ *contrast imaging* ” *OR* “ *diagnostic imaging* ”

A figura 4 apresenta as principais empresas e instituições que depositaram patentes nas famílias relacionadas às preparações radiofarmacêuticas.

**Figura 4:** Comparação entre as dez empresas que mais depositaram patentes no Brasil (BR), nos Estados Unidos (EU) e em países participantes da Convenção Europeia de Patentes (CEP), no período de 1997 a 2017, referente às famílias A61K51/00 e A61K49/00 acrescidas das palavras-chaves “ *radiopharma* ” *OR* “ *radioisotop* ” *OR* “ *radioimmun* ” *OR* “ *radioactive* ” *OR* “ *radiotherap* ” *OR* “ *radiolabel* ” *OR* “ *radionuclide* ” *.* Busca realizada pelo programa Questel Orbit©

## DISCUSSÃO

Na família A61K51/00 *,* observou-se o predomínio dos Estados Unidos no depósito de patentes, com crescimento acentuado nos últimos 10 anos. Assim como nos Estados Unidos, o número de patentes depositadas nos países da CEP é superior ao de patentes depositadas no Brasil, porém a CEP apresentou diminuição no ritmo de crescimento nos últimos 5 anos, caracterizada pela tendência a platô. O número total de patentes acumuladas pelo Brasil nesta categoria, no período observado, foi de 30.693, e o número total de patentes da CEP foi de 48.933 e dos Estados Unidos de 74.946, ou seja, o número de patentes acumuladas registradas nos Estados Unidos nesta categoria é, aproximadamente, duas vezes maior que o registrado no Brasil.

Sobre os depósitos acumulados de patentes referentes à família A61K49/00, observa-se maior número de patentes acumuladas depositadas pela CEP, seguida dos Estados Unidos e do Brasil. Enquanto a família A61K51 parece representar melhor as patentes relacionadas aos radiofármacos, incluindo os produtos que chegaram a obter registro junto às agências sanitárias regulatórias, a família A61K49 aparentemente relaciona-se, de maneira geral, aos compostos de experimentação, com potencial para aplicação *in vivo* . Nesta família, à semelhança do que foi observado para a categoria representada na figura 2A, é notória a diminuição no ritmo do crescimento nos últimos anos, com tendência a platô.

Na família A61K31/00, que compila os dados de medicamentos convencionais (não radioativos), o número de depósitos acumulados de patentes foi dez vezes maior do que o das famílias relacionadas aos processos que envolvem radiofármacos, também no caso do Brasil, com aproximadamente 310.095 patentes depositadas. O número de depósitos de patentes acumuladas pelos Estados Unidos (738.891 depósitos) é próximo do de depósitos da CEP (693.594 depósitos), no período de observação. Além disso, nesta família pesquisada, o ritmo foi crescente e sem caracterização de platô.

Por outro lado, os resultados da classificação A61K31/00, que se restringe aos medicamentos do tipo agentes de contraste radiológico usado em diagnóstico por imagem, tendem à saturação nos Estados Unidos, no Brasil e nos países da CEP.

Ao se compararem os depósitos acumulados de patentes no Brasil e nas demais regiões incluídas neste estudo, o número de depósitos foi menor no Brasil, em todas as famílias.^14^ Tal realidade é, provavelmente, reflexo, em grande parte, do nível geral de desenvolvimento do país, quando comparado aos Estados Unidos e aos países da CEP, posto que o Brasil conta com investimentos limitados por parte do governo e da iniciativa privada para desenvolvimento de novos medicamentos, incluindo os radiofármacos. Ainda, há fatores diretamente relacionados ao processo de patenteamento brasileiro: falta de apoio aos pesquisadores no processo de elaboração de patentes e questões culturais relacionadas ao incentivo à proteção da Propriedade Intelectual gerada. Essas questões podem contribuir com a desmotivação dos pesquisadores brasileiros a depositarem patentes. Outro fator a ser considerado refere-se às dificuldades encontradas no processo de depósito e ao exame de uma patente no INPI. Esse aspecto foi considerado na resolução/INPI/PR 217, de 3 de maio de 2018, que alterou a resolução 80/2013, com o objetivo de disciplinar a priorização do exame de pedidos de patentes, e patentes de produtos e processos farmacêuticos, bem como equipamentos e materiais relacionados à saúde pública.^15 , 16^

O pequeno número de depósitos de patentes que permeia a área radiofarmacêutica no Brasil poderia ser justificado pelo fato da área ser pequena no país, quando comparada à dos medicamentos em geral; pele atuação restrita aos institutos da CNEN para produção e comercialização dos radiofármacos de meia-vida longa; e pela abertura relativamente recente para a iniciativa privada, após a quebra parcial do monopólio para produção e comercialização de radiofármacos de meia-vida curta (inferior a 2 horas).^12^ Além disso, o número de pesquisadores dedicados à área radiofarmacêutica no Brasil é consideravelmente reduzido. Apesar de tais características restritivas, curiosamente, é possível constatar que o número de depósitos acumulados de patentes brasileiras relacionadas à Radiofarmácia é dez vezes menor do que na área de medicamentos em geral, esta proporcionalidade é semelhante à observada nos países mais desenvolvidos.

A pesquisa de novos medicamentos requer investimentos altos, de modo a custear todas as fases do desenvolvimento, desde a concepção da molécula ou princípio ativo, passando pelo processo farmacotécnico e das metodologias empregadas no controle de qualidade do produto acabado, estudos pré-clínicos e clínicos de Fase I a IV.^17^ O ciclo de desenvolvimento de novos radiofármacos é idêntico ao dos medicamentos convencionais. Apesar do mercado para novos radiofármacos ser pequeno, quando comparado ao dos medicamentos convencionais, os custos são comparáveis. Este fato certamente explica o menor número de depósitos de patentes na área radiofarmacêutica, quando comparado à área de medicamentos em geral, tanto no Brasil como em outros países.^18^

A regulamentação sanitária para produção e registro de radiofármacos é relativamente recente no Brasil, tendo sido implantada em 2009, a partir da publicação das RDC 63, atual RDC 301/2019 e RDC 64/2009, em consulta pública, para Boas Práticas de Fabricação e Registro de Radiofármacos, respectivamente.^11 , 19^ Tais regulamentações se fizeram necessárias, principalmente quando se consideram a quebra do monopólio de produção de radiofármacos ocorrida em 2006 e a entrada da iniciativa privada no mercado de radiofármacos.^12^

Entretanto, a RDC 64, que descreve a necessidade da realização de estudos não clínicos e clínicos para o registro de um radiofármaco inovador, impõe a esta categoria de medicamentos regras semelhantes às adotadas ao registro de medicamentos convencionais, de modo a garantir a eficácia e a segurança do produto. Exigências regulamentares deste nível também são observadas nos diferentes países e, certamente, colaboraram para frear o desenvolvimento e o lançamento de novos radiofármacos no cenário mundial, a medida em que aumentam muito o custo desse processo.^20 - 22^ A consulta pública possibilitará alterar os critérios de realização dos estudos não clínicos e clínicos.

Se, por um lado, o desenvolvimento de novos medicamentos conta, na maior parte, com o investimento de empresas de grande porte na área farmacêutica, que utilizam-se das patentes para proteger o capital investido, no caso dos radiofármacos, na maioria dos países, o desenvolvimento é normalmente realizado nos centros de pesquisa universitários ou institutos de pesquisa, para produção e uso local, em pequena escala e com dificuldades para garantir a proteção patentária do novo radiofármaco. Por outro lado, a perspectiva de transferência da tecnologia para empresas produtoras constituídas parece ser realidade cada vez mais presente na área de radiofarmácia.^23 , 24^

Por fim, as dez principais empresas e instituições que depositaram patentes nas famílias pesquisadas relacionadas às preparações radiofarmacêuticas tiveram resultados que as colocam como as responsáveis pelo maior número de depósitos de patentes, apesar da contribuição de algumas universidades − provavelmente como efeito dos altos custos envolvidos na pesquisa de novos medicamentos.^25^ Os depósitos de patentes no Brasil não refletem o desenvolvimento interno, mas a tendência de mercado, ou seja, o interesse comercial da empresa internacional, especialmente de origem americana, levando-se em consideração o custo-benefício para dada inovação.

## CONCLUSÃO

A crescente tendência dos países gerarem conhecimento técnico-científico, cada qual em seu ritmo de desenvolvimento, leva à necessidade de garantir seus direitos e estimular investimentos em determinada área. Assim, é possível visualizar a importância de um pesquisador ser assertivo na escolha e na condução de estudos experimentais, buscando processos inovadores.

O maior número de depósitos de patentes no Brasil, relacionados à área radiofarmacêutica, corresponde aos pedidos de patentes internacionais via *Patent Cooperation Treaty* , da mesma forma como os depósitos de patentes de medicamentos convencionais e/ou contrastes para radiodiagnóstico. Esse cenário é reflexo das condições de desenvolvimento econômico e científico do país, bem como das dificuldades apresentadas no processo de depósito de uma patente, mostrando ser imprescindível investir em programas que facilitem o processo e aproximem os institutos de pesquisa e as universidades da iniciativa privada, estimulando o depósito de patentes, de forma geral, e a inovação.
